# Integrin Beta 1 Promotes Glioma Cell Proliferation by Negatively Regulating the Notch Pathway

**DOI:** 10.1155/2020/8297017

**Published:** 2020-09-15

**Authors:** Weijie Min, Chao Zou, Dongwei Dai, Qiao Zuo, Chao Chen, Jinyu Xu, Yanan Li, Zhijian Yue

**Affiliations:** Department of Neurosurgery, Changhai Hospital, Second Military Medical University, 168 Changhai Road, Shanghai 200433, China

## Abstract

In this study, genes associated with the Notch signaling pathway in gliomas were analyzed using bioinformatics and in vitro experiments. The dataset GSE22772 was downloaded from the Gene-Cloud of Biotechnology Information database. Differentially expressed genes (DEGs) between short hairpin RNA (shRNA) intervening glioma cells and control cells were screened using the unpaired *t* test. Functional enrichment analysis was performed, and coexpression network was analyzed to identify the most important genes associated with the Notch signaling pathway. Integrin beta 1 (ITGB1) mRNA and protein levels in clinical glioma tumor samples and tumor adjacent normal tissue samples were analyzed using quantitative real-time PCR and immunohistochemistry, respectively. The relationship between ITGB1 expression and the prognosis of patients with gliomas was analyzed using the Kaplan-Meier curve. ITGB1 interference expression cell line U87 and ITGB1 overexpressing cell line were established using sh-ITGB1 and oe-ITGB1 plasmids, respectively. MTT and colony formation assays were used to detect changes in the proliferation of glioma cells. Moreover, western blotting was used to detect the expression of Notch and Hey1. A total of 7,962 DEGs were screened between shRNA intervening glioma cells and control cells, which were mainly associated with spliceosome, proteoglycans in cancer, focal adhesion, and the Notch signaling pathway. ITGB1 showed the highest expression in the coexpression network. The mRNA and protein expression of ITGB1 in glioma tumor samples was significantly higher than that in tumor adjacent normal tissue samples (*p* < 0.05). Overall survival time of patients in the ITGB1 low-expression group was significantly longer than that in the ITGB1 high-expression group, indicating that ITGB1 expression negatively correlated with the prognosis. Fluorescence microscopy, qRT-PCR, and western blotting confirmed the transfection efficiency of ITGB1 overexpression and interference expression in U251 and U87 cells. The MTT and colony formation assays indicated that U87 cell proliferation was significantly inhibited after intervention with ITGB1 (*p* < 0.05), and overexpression of ITGB1 significantly promoted U251 cell proliferation (*p* < 0.05). In addition, the expression of Notch and Hey1 proteins was significantly decreased after ITGB1 intervention (*p* < 0.05), and their expression was significantly upregulated after ITGB1 overexpression (*p* < 0.05). ITGB1 expression in glioma tissues was significantly higher than that in adjacent normal tissues and was negatively correlated with the survival time of patients. Therefore, ITGB1 can significantly promote proliferation of glioma cells via feedback regulation of the Notch signaling pathway.

## 1. Introduction

Malignant gliomas represent the most common type of brain tumor in adults, with an annual incidence of 5 per 100,000 individuals [1]. The overall survival of patients with glioblastoma is approximately 1 year because of its high invasiveness and rapid cell proliferation [2]. Certain genes play important roles in glioma diagnosis and treatment, such as isocitrate dehydrogenase 1 (IDHl) [3], and mutations of such genes result in promoter methylation of O6-methylguanine-DNA methyltransferase (MGMT) and 1p/19q deletion [4, 5]. Though three types of biological markers have been adopted for the diagnosis and treatment of gliomas [6–8], subtyping function of the known markers was still unable to meet the needs of researchers and clinicians. Therefore, more important pathogenic genes need to be explored to develop novel diagnostic and therapeutic strategies for gliomas.

Recently, the role of the Notch signaling pathway in the development of glioma has aroused great interest of researchers. The Notch signaling pathway is essentially involved in the maintenance of a variety of adult stem cells [9], and aberrant Notch activity is found in a wide range of human tumors, including glioma [10]. Overexpression of Notch-1 and its ligands, Delta-Like-1 and Jagged-1, is critical for glioma cell survival and proliferation [11], and activation of Notch via expression of NICD1 promotes growth and neurosphere formation of the SHG-44 glioma cell line [12]. Recent results have indicated that oroxylin A exhibits antimalignant glioma proficiency by inducing autophagy via the ERK/AKT-mTOR-STAT3-Notch signaling cascade [13]. Considering Notch's function in stem cell and cancer biology, we believe that screening genes closely associated with the Notch signaling pathway will provide important information for the development of diagnostic and treatment strategies for gliomas.

Mammalian genome contains four Notch homologous genes, which were bound with five ligands (Deltex 1/3/4, Jagged 1/2). Deltex-1 (DTX1) has been identified as an E3 ubiquitin ligase, which serves as an important signaling component downstream of Notch that regulates transcription of target genes in the nucleus [14–18]. Huber et al. reported that overexpression of DTX1 significantly promoted the invasiveness of glioma cells, whereas patients with low expression of Deltex-1 had a relatively good prognosis [19].

In this study, we tried to screen genes associated with the Notch signaling pathway in malignant gliomas using bioinformatics analysis. The results were validated in the clinical samples, and their mechanism of action was further explored in vitro.

## 2. Materials and Methods

### 2.1. Dataset

The datasets were screened with “glioma” and “Notch signaling pathway” as key words in the Gene-Cloud of Biotechnology Information database (GCBI). The selection criteria included (1) *Homo sapiens* as the sample source, (2) RNA chip or sequencing data, (3) more than six samples, and (4) alterations in the Notch signal pathway. Finally, the dataset GSE22772 in which small hairpin (sh) RNA was used to interfere with Deltex-1 expression to activate the Notch signaling pathway in the glioma cell line U373 was selected.

### 2.2. Bioinformatics Analysis

#### 2.2.1. Differentially Expressed Genes

The differentially expressed genes (DEGs) between shRNA intervening glioma cells and control cells were screened using the unpaired *t* test. The *Q* value <0.04 and fold change (FC) >1.1 were set as thresholds.

#### 2.2.2. Functional Enrichment Analysis

Gene ontology (GO) analysis and Kyoto Encyclopedia of Genes and Genomes (KEGG) enrichment analysis were performed for the DEGs in Database for Annotation, Visualization, and Integrated Discovery (DAVD, https://david.ncifcrf.gov/) with *p* < 0.05 as threshold.

#### 2.2.3. Coexpression Network Analysis

The DEGs included in top 10 enriched GO terms and KEGG pathways were used to establish the coexpression network. The coexpression correlations of the mRNAs were identified in the STRING database (https://string-db.org/) [20]. The interaction network was established using the interaction pairs with an interaction score above 0.8, which was later visualized using Cystoscope software.

#### 2.2.4. Clinical Samples and Follow-Up

The present study was approved by the Institution Review Broad of the Changhai Hospital, Shanghai. Written informed consent was obtained for each patient. All experiments were performed in accordance with the approved guidelines. The glioma tissue and paired peritumoral tissues were collected in the Department of Neurosurgery of Changhai Hospital, which were rapidly frozen in liquid nitrogen after surgical removal and then stored at −80°C until further use.

Samples for quantifying ITGB1 mRNA and protein expression were collected from December 2014 to September 2015. The criteria for inclusion of clinical cases were as follows: (1) the pathological diagnosis being glioblastoma or astrocytoma and (2) the tumor located in the convex surface of the brain. The criteria for exclusion included the following: (1) glioma located in other parts of the brain, cerebellum, spinal cord, etc., (2) the interval of collection time between tumor and peritumoral tissue more 30 min, and (3) the samples not preserved in −80 °C or transported in liquid nitrogen.

Samples for the correlation analysis between the expression level of ITGB1 and prognosis were collected from January 2010 to December 2013. The criteria for inclusion were as follows: (1) the pathological diagnosis higher than grade III (Diamandis Phedias and Kenneth Aldape, 2018), (2) the tumor located in the convex surface of the brain, and (3) total incision performed under the surgical microscope. The criteria for exclusion included the following: (1) multiple intracranial lesions, (2) presence of other congenital diseases or serious basic diseases in addition to glioma, (3) other causes leading to the end of the event, (4) patients with severe complications, such as intracranial infection or intracranial hematoma after surgery, and (5) scarcity of samples or poor sample quality. The outpatient follow-up data of the patients recruited for the study were collected and telephone follow-up was conducted to collect the patient's overall survival information.

#### 2.2.5. Quantitation of ITGB1 mRNA Expression in Glioma Tissue

Total RNA of sample tissue was isolated using TRIzol (Invitrogen, Carlsbad, USA), and cDNA was synthesized using the PrimeScript RT reagent kit (Takara, Dalian, China) following the manufacturer's instructions. Quantitative real-time PCR (qRT-PCR) was carried out using the SYBR ® Premix ExTaq kit (Takara, Dalian, China) and the 7300 Real-Time PCR Detection system (ABI, USA). The primers of ITGB1 and GADPH used for qRT-PCR were synthesized by Sangon Biotech (Shanghai, China). The primer sequences are shown in Table 1.

#### 2.2.6. Immunohistochemistry of ITGB1 Protein Expression in Glioma Tissue

After fixing with 4% paraformaldehyde, the excised tissue was soaked in 20% sucrose solution overnight at 4°C. Continuous paraffin sections were cut and transferred onto glass slides, which were treated by 0.3% hydrogen peroxide-methanol solution and 0.3% Triton X-100 for 30 min. The sections were immersed in Integrin *β*1 (D2E5) rabbit mAb antibody (CST, Danvers, USA) with 1 : 400 at 4°C overnight, followed by anti-rabbit IgG, HRP-linked antibody (CST, Danvers, USA) with 1 : 100 dilution at room temperature for 2 h. Immunohistochemical images were analyzed using the Allred scoring system [21, 22]. Proportion of complete membranous stained cells was recorded in four categories including (1) negative (−), <1%, (2) first-degree positive (+), 1–10%, (3) second-degree positive (++), 10–50%, and (4) third-degree positive (+++), ≥50%. The staining intensity is divided into weak, medium, and strong. If the intensity is weak, the positive grade is reduced by one level.

#### 2.2.7. Quantitation of ITGB1 mRNA Expression in Glioma Cell Lines

Five human glioma cell lines, U87, U251, U373MG ATCC, SHG44, and T98G, which were authenticated by STR profiling, were acquired from Changhai Hospital. The cells were cultured in Dulbecco's Modified Eagle Medium (DMEM) supplemented with 10% fetal bovine serum (Invitrogen). All cells used in the experiments were harvested in the logarithmic growth phase. The harvested cells were washed with PBS, and then 1 ml Trizol was added and kept at room temperature for 5 min. The isolation of total RNA, the synthesis of cDNA, and qRT-PCR were performed as previously described.

#### 2.2.8. Construction of ITGB1 Interference Expression and Overexpression of Cell Lines

The lentiviral vector containing the primers for interference expression and overexpression of ITGB1 (Table 2) was purchased from GenePharma (Suzhou, China). After filtration through a 0.5 *μ*m microfiltration membrane, 1 ml of lentiviral medium and 1 ml of virus solution were added to U251 and U87 cells and cultured in an antibiotic-free medium for 24 h at a density of approximately 30%. The final concentration of virus particles was 4 *µ*g/ml polybrene.

#### 2.2.9. Detection of ITGB1 Expression Level Using qRT-PCR and Western Blot

After transfecting U87 and U251 cells with sh-ITGB1 and oe-ITGB1 virus for 48 h, total RNA was extracted, and the ITGB1 mRNA expression level was detected by qRT-PCR. In addition, 72 h after transfection, total protein was extracted by IP lysate containing benzyl sulfonyl fluoride (Pierce) and ultrasonic breakage was performed for western blotting. ITGB1 protein concentration was determined by the Coomassie brilliant blue method. Western blotting was performed according to the standard procedure. In brief, the samples were electrophoretically transferred to a PVDF membrane after SDS-PAGE. The membrane was blocked with 1% BSA at room temperature for 1 h and then incubated with mouse anti-ITGB1 antibody (1 : 1,000, Abcam) overnight at 4°C, followed by incubation with horseradish peroxidase-conjugated goat antimouse IgG (1 : 5,000, Abcam) at room temperature for 1 h. The membranes were washed three times with PBST between each step. GAPDH was used as an internal reference protein.

#### 2.2.10. MTT Assay

Glioma cells in the logarithmic phase were plated in 96-well plates at 5 × 10^3^ per well and cultivated. After the bottom of the hole was filled with the cell monolayer for 6 h, the cells were transfected with the viral plasmid. After 3-day culture, 3-[4,5-dimethylthiazol-2-yl]-2,5-diphenyltetrazolium bromide (MTT, 5 mg/ml, Invitrogen) was added and cultivated at 37°C for 4 h. Then, the culture was disposed, by adding 150 *μ*l dimethylsulfoxide (DMSO, Invitrogen) in each well. Absorbance was measured at 490 nm. The data were recorded as mean ± standard deviation. The *t* test was used to compare the data between two groups, and *p* < 0.05 was set as the threshold.

#### 2.2.11. Colony Formation Assay

Glioma cells in the logarithmic phase were added in the DMEM medium (supplemented with 10% FBS) to make single cell suspension. After gradient dilution of cell suspension, 50, 100, and 200 cells were plated in preheated DMEM medium (supplemented with 10% FBS) per well, respectively. They were cultured for about 14–21 days at 37°C, 5% CO_2_, and saturated humidity. The culture was terminated when clones were visible. Cells were fixed with 4% polyoxymethylene for 15 min and were dyed with Giemsa staining solution for 20 min. The number of the clones was assessed by counting under a microscope, and clone formation rate was also evaluated. The clone formation rate refers to the percentage of the number of clones that account for the total number of inoculated cells.

#### 2.2.12. Detection of Notch and Hey1 Protein Expression Using Western Blot

After ITGB1 expression was interfered and overexpressed, the expression of Notch and Hey1 proteins was detected using western blotting. The mouse anti-Hey1 antibody, Notch antibody, and GAPDH antibody were purchased from Abcam. The rest of the protocol was the same as that of ITGB1 protein detection.

## 3. Results

### 3.1. DEG Screening and Functional Enrichment Analysis

**Figure 1:**
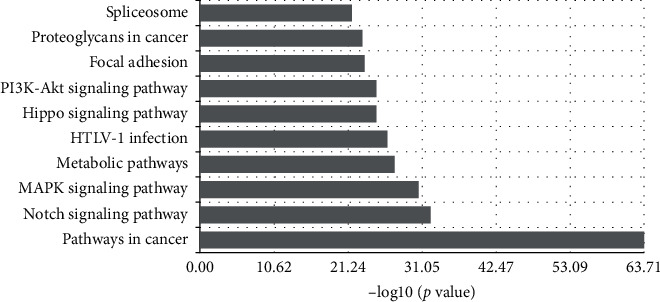
Enriched KEGG pathways of differentially expressed genes.

**Figure 2:**
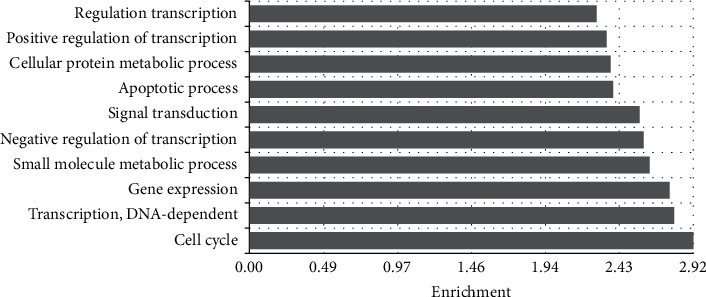
Enriched GO terms of differentially expressed genes.

A total of 7,962 DEGs were screened between shRNA intervening glioma cells and control cells with the criteria of *Q* value < 0.04 and fold change >1.1. Functional analysis was performed to reveal the enriched GO terms and KEGG pathways. Top 10 enriched KEGG pathways included spliceosome and proteoglycans in cancer, focal adhesion, PI3K-Akt signaling pathway, Hippo signaling pathway, HTLV-I infection, metabolic pathways, MAPK signaling pathway, Notch signaling pathway, and pathways in cancer (Figure 1). Top 10 enriched GO terms included transcriptional regulation, positive transcription regulation, cellular protein metabolism, apoptosis process, signal transduction, negative transcription regulation, small molecule metabolic processes, gene expression, DNA-dependent transcription activity, and cell cycle (Figure 2).

### 3.2. Coexpression Network Analysis

**Figure 3:**
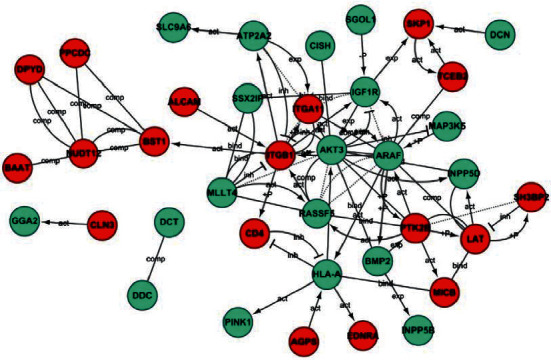
Gene coexpression network. Red circles represent upregulated genes and relatively blue circles represent downregulated genes.

The coexpression network of DEGs in shRNA intervening glioma cells was established (Figure 3). The red and green circles represent upregulated and downregulated genes, respectively. In the network, ITGB1 showed the highest degree of correlation (Table 3), suggesting that ITGB1 may be one of the most important factors associated with the Notch signaling pathway.

### 3.3. Expression ITGB1 in Glioma Samples

**Figure 4:**
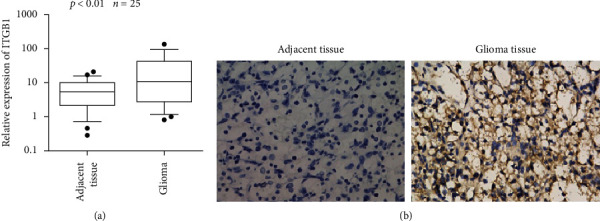
ITGB1 expression at the mRNA (a) and protein (b) level in gliomas and peritumoral normal tissues.

Twenty-five glioma tissue samples and corresponding peritumoral normal tissues were collected, of which one pair of sample was excluded because of poor sample quality. Remaining 24 pairs of samples were used to detect the expression level of ITGB1. qRT-PCR revealed that the average mRNA expression levels of *ITGB1* in glioma samples were significantly higher than those in the peritumoral normal tissues (*p* value = 0.0022) (Figure 4(a)).

Immunohistochemical staining showed that the ITGB1 positive expression rate in glioma cells was 85.7% with strong staining intensity (+++), while the positive expression rate in peritumoral normal tissues was 14.3% with the weak staining intensity, which was determined as one grade decline, suggesting ITGB1 negative expression (−). The expression level of ITGB1 protein in gliomas was significantly higher than the expression level in adjacent normal tissue (*p* value = 0.014) (Figure 4(b)).

### 3.4. Correlation between ITGB1 Expression and Prognosis of Gliomas

**Figure 5:**
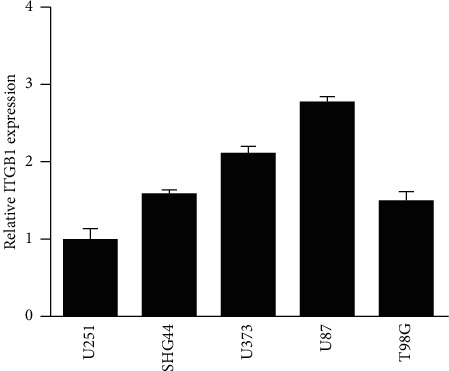
ITGB1 expression in five glioma cell strains.

According to the medical records of Changhai Hospital from January 2010 to December 2013, a total of 267 patients with gliomas underwent surgery, 43 of them followed up. The *ITGB1* expression levels in the glioma tissues of the 43 patients were investigated by qRT-PCR (data not shown). According to the median expression level of ITGB1 in all tumor tissues, the 43 samples were divided into ITGB1 high-expression group (H) and ITGB1 low-expression group (containing median, L), for which clinical features are listed in Table 4. There was no significant different in age, sex, and pathological grade between the H and L groups (*p* > 0.05). The relationship between *ITGB1* expression level and glioma prognosis was analyzed in the 43 patients. Kaplan-Meier (KM) curve analysis showed that the overall survival time (OS) of patients in the L group (32.90 ± 4.12 months) was significantly longer than OS in the H group (14.60 ± 2.08 months) (*p* < 0.001) (Figure 5), indicating that expression level of *ITGB1* was negatively correlated with the prognosis of glioma.

### 3.5. Interference Expression and Overexpression of ITGB1

**Figure 6:**
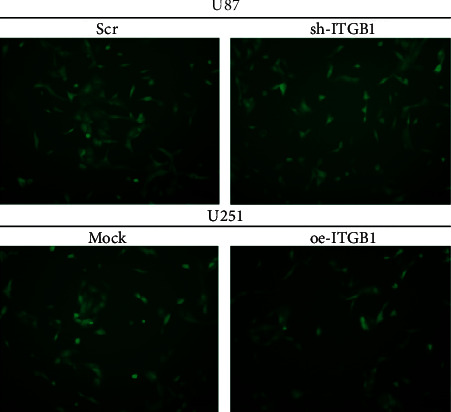
Transfection efficiency of glioma cell strains U87 and U251 transfected with shRNA-ITGB1 and oe-ITGB1, respectively.

The *ITGB1* expression levels were firstly detected in five glioma cell lines, U87, U251, U373, SHG44, and T98 G using qRT-PCR. The lowest and highest expression levels of *ITGB1* were found in U251 and U87, respectively (Figure 6). Therefore, U251 and U87 cell strains were selected for further study.

**Figure 7:**
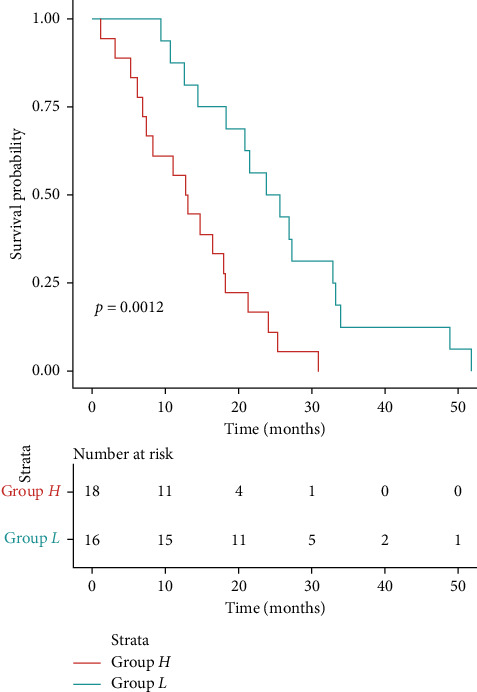
Kaplan-Meier analysis of the expression level of ITGB1 and prognosis of glioma patients.

**Figure 8:**
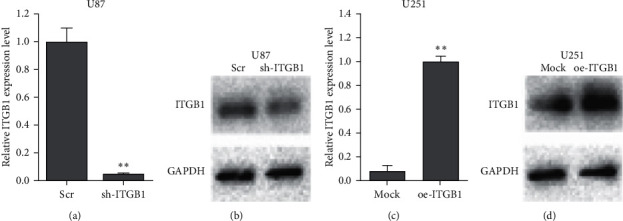
The efficiencies of interference and overexpression of ITGB1 observed by qRT-PCR and western blotting in the glioma cell lines U87 (a, b) and U251 (c, d) after transfection with sh-ITGB1 and oe-ITGB1, respectively; *∗∗p* < 0.05.

Lentivirus was transfected into U87 cells to interfere with the *ITGB1* expression, and overexpressing lentivirus was transfected into U251 cells. The average transfection efficiencies of both interference and overexpression reached to 80% (Figure 7), suggesting ITGB1 interference expression and overexpression cell lines were successfully constructed. The results showed that shRNA-ITGB1 significantly decreased the expression of ITGB1 at both transcriptional and translational levels in the ITGB1 interference group of U87 cells (Figures 8(a) and 8(b)). On the contrary, both mRNA and protein of ITGB1 had significantly high expression patterns (*p* < 0.05) in the ITGB1 overexpression group of U251 cells (Figures 8(c) and 8(d)).

Therefore, lentivirus-mediated sh-ITGB1 transfection suppressed the *ITGB1* expression in the U87 cell line. Similarly, lentivirus-mediated ox-ITGB1 transfection overexpressed *ITGB1* in the U251 cell line. The effect of ITGB1 expression on glioma proliferation and the association between ITGB1 and Notch signaling pathway were further explored.

### 3.6. ITGB1 Promotes the Glioma Proliferation

**Figure 9:**
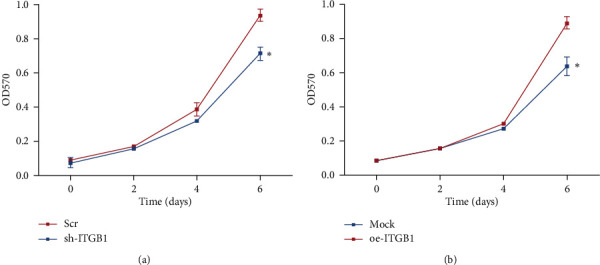
Changes in the proliferation ability of glioma cells U87 (a) and U251 (b) with interfered and overexpressed ITGB1, respectively.

MTT results indicated that there was no significant change in the proliferation ability of U87 cells between the control group and the experimental group for the first four days after the downregulation of ITGB1. However, the proliferation rate of glioma cells in the interference group started from the fourth day and was significantly lower than that in the control group until the sixth day (*p*=0.025) (Figure 9(a)). The trend of U251 cell proliferation ability between the control group and the experimental group was similar to that in U87 cell during the first four days after overexpression of ITGB1. The proliferation rate was significantly higher in ITGB1 overexpression glioma cells than that in the control group from the fourth day to the sixth day (*p*=0.018) (Figure 9(b)).

**Figure 10:**
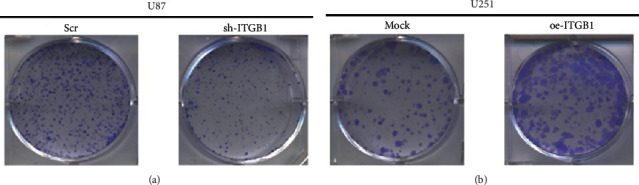
Effects of ITGB1 interference and overexpression on clone formation of glioma cells U87 (a) and U251 (b), respectively.

The clone formation assay further verified the effects of ITGB1 on the proliferation of glioma cells. The proliferation ability of U87 cells was significantly lower than that of the control group after the downregulation of ITGB1 (*p*=0.033) (Figure 10(a)), while the proliferation ability of U251 cell with overexpression of ITGB1 was significantly higher compared with the control group (*p*=0.029) (Figure 10(b)).

### 3.7. Relationship between ITGB1 and the Notch Pathway

**Figure 11:**
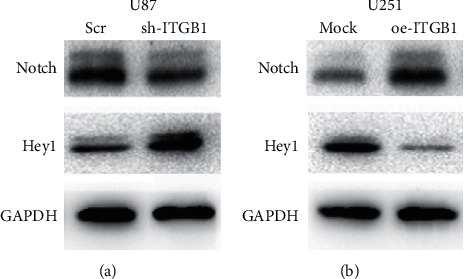
Effects of interference and overexpression of IGTB1 on the expression levels of Notch (a) and Hey1 (b) proteins.

In order to validate the relationship between ITGB1 and the Notch pathway, the expression levels of Notch and Hey1 proteins in U87 and U251 cells were detected after their ITGB1 genes were interfered and overexpressed, respectively. The expression of Notch and Hey1 proteins decreased significantly after ITGB1 expression was interfered (Figure 11(a)). In contrast, transcripts of the two proteins increased significantly after ITGB1 was overexpressed in U87 and U251 cells (Figure 11(b)). These results suggested that ITGB1 activated the Notch pathway.

## 4. Discussion

Recent studies have shown that the Notch signaling pathway is closely associated with the occurrence, development, invasiveness, and angiogenesis of glioma. For example, the interference of Notch1 in glioma cells can promote the apoptosis, proliferation, and cell cycle arrest of glioma cells [11]. As a ligand for Notch nonclassical pathways, DTX1 has a negative effect on Notch signaling pathway [14–17]. In this study, the GSE22772 chip data with interfering DTX1 expression were downloaded from the GCBI database, and the changes in Notch signaling pathway were analyzed. A total of 7,962 DEGs were identified. The coexpression network was constructed and analyzed. It was seen that the interference of DTX1 in glioma cells can activate the Notch signaling pathway and then produce a series of regulatory effects. Top 10 enriched signaling pathways were mainly involved in regulating cell apoptosis, cell cycle, and signal transduction, which was consistent with the previous studies [11].

ITGB1 is a member of the integrin family, beneficial to survival, differentiation, angiogenesis, and invasion of cancer cells mediated by the interaction between cells and extracellular matrix [23–26]. It has previously been suggested that ITGB1-mediated signaling essentially contributed to cell survival after radiation-induced genotoxic injury [27], and increased expression of integrin *β*1 was associated with poor prognosis in patients with pancreatic carcinoma [28], small-cell lung cancer [29], invasive breast cancer [30], and multiple myeloma [31]. Previous study has indicated that ITGB1 immunostaining was heterogeneous in both intensity and frequency, and its distribution was patchy throughout the glioblastoma tumor sample with strongly positive immunostaining signal often close to the necrotic regions [32]. Considering its important role in chemotherapy and radiation resistance, *β*1-integrin might be an important target for antitumor therapy [27, 31, 33]. However, as a prognostic indicator, the relevance and clinical significance of ITGB1 and glioma are rarely mentioned. In this study, ITGB1 was shown as one of the most important genes related to the Notch signaling pathway in glioma, affecting the expression of the other genes.

The relationship between ITGB1 expression in glioma tissues and their prognosis was analyzed in clinical samples. The result indicated that the expression level of ITGB1 in glioma tumor tissues is significantly higher than that in the normal tissue. The increased expression of ITGB1 was also observed in other tumors, such as prostate cancer [34], ovarian carcinoma [35], lung cancer [36], colorectal adenocarcinomas [37], and in triple negative breast cancer [38]. Moreover, the expression of *β*1-integrin protein was significantly higher in colorectal adenocarcinomas tissue samples of stage III than those in stage I-II [37], indicating that the expression level of *ITGB1* might be associated with the stage of disease.

The reliability of ITGB1 as a potential biomarker was further explored, and the relationship between ITGB1 expression and prognosis of 43 glioma patients was analyzed. It was found that the patients with low *ITGB1* expression had longer survival time than those with high *ITGB1* expression, suggesting that ITGB1 was negatively correlated with the prognosis of glioma. This was consistent with the results of other tumors, which indicated that increased expression of integrin *β*1 was associated with poor prognosis of patients with small-cell lung cancer [39], invasive breast cancer [30], and multiple myeloma [31]. The high expression level of ITGB1 in glioma tumor tissues and its correlation with the clinical prognosis indicated that ITGB1 might play an important role in the development of glioma tumor and suggested ITGB1 to be a potential marker for glioma prognosis.

The mechanism of how ITGB1 functioned in glioma was explored in this study. High proliferation and strong invasiveness are important reasons for the poor prognosis of glioma [2, 40], and ITGB1 is often expressed abnormally in cancer and correlates with malignant tumor phenotypes, such as invasion, migration, angiogenesis, and proliferation [41]. Therefore, we speculated that ITGB1 might affect the proliferation of glioma. To test this hypothesis, we altered the expression level of ITGB1 by interference and overexpression and examined the proliferation and colony formation ability of glioma cells. The results of MTT experiment and clone proliferation experiment showed that ITGB1 could increase the cell activity and promote cell proliferation. This was consistent with the results obtained for other cancers, which suggested that linc-ITGB1 could promote the invasion and migration of gallbladder cancer cells [42], and high expression of ITGB1 could increase the invasion of breast cancer cells [38]. It is also reported that ITGB1 can decrease the adhesiveness between tumor cells, promote the detachment of tumor cells from the tumor body, and enhance adhesion between tumor cells and the ECM [43].

Considering the important role of ITGB1 in the Notch signaling pathway, the relationship between ITGB1 and the Notch pathway was further explored. We detected changes in the expression of Notch and Hey1 proteins after interference or overexpression of ITGB1. The results indicated that ITGB1 could positively regulate the expression of Notch and Hey1 proteins. Previous studies have shown that not only the Notch pathway but also the expression of ITGB1 could be activated by hypoxia [44, 45]. The hypoxia-inducible factor 1*α* (HIF1*α*) can directly act on the promoter of ITGB1 and activate the transcriptional expression of ITGB1 [44]. We speculated that the Notch signaling pathway was activated due to other reasons. For example, the excess unbound HIF1*α* can activate ITGB1, which may be the mechanism by which HIF1*α* activation regulates Notch. However, the specific process by which ITGB1 regulates the Notch signaling pathway needs more research to confirm.

Despite the above results, there are some limitations of this study. (1) In the relationship analysis between ITGB1 expression and gliomas' prognosis, the effects of homogenous treatment factors, not associated with ITGB1 expression, on survival should be ruled out as much as possible, such as tumor sites, tumor resection, multiple tumors, and postoperative complications. (2) The model for evaluating the relationship between ITGB1 expression and survival in glioma patients may be too simple to fully reflect the actual situation. In addition, it should be noted that all cases do not include low-grade gliomas. (3) Although ITGB1 of high-grade gliomas was verified to be independent of pathological grading by the modified the chi-squared test, the ITGB1 expression among different classes of low-grade gliomas and between high-grade and low-grade gliomas could not be interpreted due to limited sample. (4) There is no experiment to explore the mechanism of the Notch pathway promoting the migration and invasion of glioma cells. (5) Some expression of ITGB1 was only verified by qPCR without WB. Therefore, in future studies, we should increase the number of samples to validate the relationship between ITGB1 expression and pathological grade; experiments need be to be designed to explore the mechanism of the Notch pathway promoting the migration and invasion of glioma cells, and the functional analysis of ITGB1 need to be verified at both mRNA and protein levels.

## 5. Conclusion

The expression of *ITGB1* in glioma tissues was significantly higher than that in adjacent normal tissues and was negatively correlated with the survival time of patients. ITGB1 can significantly promote the proliferation of glioma cells via feedback regulation of the Notch signaling pathway.

## Figures and Tables

**Table 1:** qRT-PCR primers for ITGB1 and GADPH.

Primer name	Sequence (5′-3′)
ITGB1-RTF	CCTTGGGATGACTTGATTG
ITGB1-RTR	ACCTTTCGGTCACTTAGGGGG
GAPDH-RTF	CTGGGCTACACTGAGCACC
GAPDH-RTR	AAGTGGTCGTTGAGGGCAATG

**Table 2:** The primers for interference expression and overexpression of ITGB1.

Primer name	Sequence (5′-3′)
ITGB1-shRNA-F	UUCUCCGAACGUGUCACGUTT
ITGB1-shRNA-R	ACGUGACACGUUCGGAGAATT
ITGB1-overexpression-F	TCATCTAGAGTTAATCAGCATG
	TCATGGCCTACCCCTACGACGT
ITGB1-overexpression-R	TCCTGCAGCCCGTAGTTTTCAG
	GTGGCCTGGTCCAG

**Table 3:** Network degree analysis of differential expressed genes.

Gene	Degree
ITGB1	11
RASSF5	9
AKT3	7
ARAF	7
PTK2B	6
HLA-A	6
IGF1R	6
LAT	5
NUTD12	5
SKP1	5
ATP2A2	4

**Table 4:** The clinical features of the 43 glioma patients.

Group	Number	Gender		Survival/death patients		Pathological grading			Average age (years)
		Male	Female	Survival	Death	III	III-IV	IV	
H	19	13	6	1	18	2	2	15	50.63
L	24	14	10	8	16	7	0	17	46.67

## Data Availability

The datasets used and analyzed during the current study are available from the corresponding author on reasonable request.
